# What Opportunities Exist for Making the Food Supply Nutrition Friendly? A Policy Space Analysis in Mexico

**DOI:** 10.34172/ijhpm.2021.164

**Published:** 2021-11-28

**Authors:** Gloria Cervantes, Anne-Marie Thow, Luis Gómez-Oliver, Luis Durán-Arenas, Carolina Pérez-Ferrer

**Affiliations:** ^1^Master´s and Doctorate Program in Medical and Health Sciences, Faculty of Medicine, National Autonomous University of Mexico, Mexico City, Mexico.; ^2^Menzies Centre for Health Policy, School of Public Health, The University of Sydney, Sydney, NSW, Australia.; ^3^Faculty of Economics, National Autonomous University of Mexico, Mexico City, Mexico.; ^4^Department of Public Health, Faculty of Medicine, National Autonomous University of Mexico, Mexico City, Mexico.; ^5^Center for Research in Nutrition and Health, National Institute of Public Health, Cuernavaca, Mexico.; ^6^National Council for Science and Technology (CONACYT), Mexico City, Mexico.

**Keywords:** Food System, Food policy, Policy Analysis, Diet, Malnutrition, Mexico

## Abstract

**Background:** As part of a global policy response for addressing malnutrition, food system actions have been proposed. Within food system interventions, policies directed to supply chains have the potential to increase the availability and affordability of a healthy diet. This qualitative study aimed to identify opportunities to integrate nutrition as a priority into the food supply policy space in Mexico.

**Methods:** Data were collected through analysis of 19 policy documents and 20 semi-structured stakeholder interviews. As an analytical framework, we used policy space analysis and embedded the Advocacy Coalition Framework (ACF) and the steps of the food chain of the conceptual framework of food systems for diets and nutrition.

**Results:** Policy issues relevant to nutrition were viewed differently in the economic and agricultural sectors versus the health sector. Overall, the main policy objective related to nutrition within the economic and agricultural sectors was to contribute to food security in terms of food quantity. Nutrition was an objective in itself only in the health sector, with a focus on food quality. Our policy space analysis reveals an opportunity to promote a new integrated vision with the recent creation of an intersectoral group working on the public agenda for a food system transformation. This newer integrative narrative on food systems presents an opportunity to shift the existing food security narrative from quantity towards considerations of diet quality.

**Conclusion:** The political context and public agenda are favorable to pursue a food system transformation to deliver sustainable healthy diets. Mexico can provide a case study for other low- and middle-income countries (LMICs) for putting nutrition at the center of food policy, despite the ongoing constraints on achieving this.

## Background

**Key Messages**

** Implications for policy makers**

- Integrating nutrition into national food policy is influenced by opportunities and constraints that must be understood to help inform policy change.
- Tensions were revealed between different national sectors (economy, agriculture and health) through this exploration of different actors’ beliefs regarding food policy and nutrition.
- A new national multisectoral working group with a vision to create a healthy, sustainable, fair and competitive food system represents an opportunity to shift the existing food security narrative towards a wider recognition of nutrition and sustainability as central aspects of food security.
- Opportunities for specific points of change in the existing food policies in Mexico include integration of nutritional criteria in the selection of crops and foods that are supported by policies, and financial and technical support for connecting small-farmers’ production to markets for commercialization of a wide range of nutrient-rich foods.

** Implications for the public**

Because malnutrition in all its forms -obesity and undernutrition- is a significant health challenge, remedying poor diets is key to population health. As part of a response, experts groups recommend food supply policy interventions directed to production and food distribution with the potential to increase the availability and affordability of healthy diets. This study identified opportunities to integrate nutrition as a priority into the food supply policy space using Mexico as a case study. The findings provide insight for strategic advocacy for policy change that links nutrition priorities with food supply actions. Findings suggest that established food security narratives of food quantity are encountering a newer policy narrative of food systems transformation. The new narrative represents an opportunity to include diet quality in the policy space that addresses population nutrition improvement.

Malnutrition in all its forms, which includes overweight, obesity, undernutrition and micronutrient deficiencies, is a significant health and development policy challenge for low- and middle-income countries (LMICs).^1-3^ In Mexico, there is persistent undernutrition coexisting with a rising prevalence in diet-related non-communicable diseases (NCDs). An undernutrition burden persists with almost 1.5 million Mexican children younger than five years with low height for age; thus, 14% of children have chronic undernutrition.^4^ At the same time, overweight-obesity prevalence is 33% in schoolchildren and 73% in adults.^4^ These trends of undernutrition and obesity reflect what is known as the double burden of malnutrition.

Although the causes underlying malnutrition are complex, poor diet makes the biggest contribution.^5,6^ In Mexico, only 42% of adults eat vegetables regularly, and 1%-4% reach the recommended intake of legumes, in contrast to 85% who drink sugar sweetened beverages regularly.^4,7^ It is not only necessary to reduce dietary risk factors for malnutrition; increased intake is required of nutrient-rich foods like vegetables and fruits, whole grains, nuts, legumes and seafood, and reduced intake is required of ultra-processed foods containing excess amounts of fat, sugar and salt.^8-11^

As part of a policy response for addressing malnutrition and NCDs globally, several international organizations and expert groups recommend multidimensional policy interventions that target both the immediate and the underlying and basic causes of malnutrition.^6,12-15^ Interventions to address the immediate causes focus on individuals’ behaviors while interventions with focus on the underlying and basic causes aim to promote healthier environments and systemic changes.^16-18^

Food system transformation has been identified as necessary, globally, to deliver healthy diets. Food supply policy actions include reorientation of agricultural priorities from producing high quantities of food to producing a wide range of nutrient-rich food, investments in nutrition-sensitive food supply chains, and focus on trade and investment for improved nutrition.^8,14,19,20^ In addition, global development agendas that are underway have undertaken nutrition-oriented goals, but most governments are currently unable to meet them.^15^ For example, the United Nations Sustainable Development Goal number two to end hunger, food insecurity and all forms of malnutrition is off track to be achieved by 2030, and Mexico is struggling to reach targets.^21^ The global difficulties are due, in part, to issues that shape agendas, including economic slowdowns, downturns and the coronavirus disease 2019 (COVID-19) pandemic which have affected the food system.^15^

To date, the Government of Mexico has adopted a range of interventions for addressing immediate and underlying causes of malnutrition, but few have been undertaken from a food system perspective. Mexico has made a significant progress in interventions such as food assistance programs, food regulations in the school environment, advertising to children, food labelling, and tax on sugar sweetened beverages.^22-30^ However, there has been limited uptake of recommended policy interventions that target the food supply. Simultaneously, the North American Free Trade Agreement (NAFTA) has been credited with furthering policies that promote an industrialized food system.^31^ Despite these nutrition-absent policies, the literature indicates that malnutrition could be ameliorated by the inclusion of nutritional considerations into multisectoral policies. Policies governing national agriculture, distribution and food trade have the potential to increase the availability and affordability of healthy diets.^8,16-19^

Challenges of integrating nutrition into multisectoral policies have been widely documented in the literature, including poor governance, lack of government effectiveness, and lack of ability and capacity to translate evidence from different disciplines into business cases beyond sectoral boundaries.^32-35^ These challenges have also been highlighted by the recent focus on food system transformations to deliver healthier diets and better nutrition, which also require engagement from a range of sectors.^19,32^ Unfortunately, nutrition itself is not a sector, but is dependent on actions that originate from a range of sectors: health, agriculture, social protection, and water, sanitation and hygiene.^32^ Policy coordination and integration across sectors have proved challenging in health policy and planning related to NCDs prevention and regulation more broadly. For example, tobacco, alcohol, and sugary drinks in LMICs encountered industry market promotion and policy interference in addressing NCDs, and evidence argues for more policy coherence and good governance in terms of multisectoral action.^33,36^ Previous research has also identified strategies and opportunities for addressing these challenges, such as consideration of external driving forces through international agencies and capacity development to generate evidence and use it to generate health literacy and good governance of a country.^33,34^ Given the severity of both forms of malnutrition in Mexico, this study provides an understanding of policy opportunities by examining in detail the multisectoral policy space for nutrition in Mexico, and specifically examines the potential to integrate nutrition into those sectors that govern the food supply.

 This study contributes to a better understanding of multisectoral food system policy for improving nutrition in LMICs – and thus to the global priority of food system transformation – through an analysis of the case of Mexico. Mexico thus presents a relevant case study for improving the understanding of constraints and opportunities to inform future food policy that links nutrition health priorities with food supply actions to deliver healthy diets. A policy space analysis approach to address both forms of malnutrition – undernutrition and obesity – in relation to the food chain has not previously been performed in Mexico.

## Methods

This qualitative study aimed to identify opportunities and constraints to integrate nutrition as a priority into the food supply policy space in Mexico. The study was performed within the existing political and policy context for nutrition. We considered political aspects as referring to the actors who make policy (within the bureaucratic and political spheres), and policy aspects as referring to statements or formal positions of intent or actions by government in the relevant sectors.^37^

###  Study Setting and Frameworks 

This case study was carried out at a national level in Mexico from August 2019 to January 2020 and included semi-structured interviews with high-positioned governmental stakeholders and document analysis. As analytical framework, we used policy space analysis ^38^ and embedded the Advocacy Coalition Framework (ACF)^39^ and the steps of the food chain of the conceptual framework of food systems for diets and nutrition.^14^

Policy space analysis has been previously used for analyses in developing countries and in public health.^3,40^ Policy space defined by Grindle and Thomas is a “space determined by the ability of a regime and its political leadership to pursue a reform measure.”^38^ The Policy Space framework focuses on the interrelation between three dimensions for policy change: context, agenda setting circumstances, and policy characteristics.^38^ The context indicates a pre-existing situation of policy sectors and characteristics of a given country such as historical, political and economic which provides a scope for opportunity for the pursuit of change. Agenda setting is the act of determining public priorities by policy actors and is influenced by their perception of a situation of a specific problem and decision-making concerns. The policy characteristics form a bridge between what was decided and the consequences that followed.^38^ This Policy Space framework underpinned the development of the interview guide and the analytical approach used in this study.

**Figure 1:**
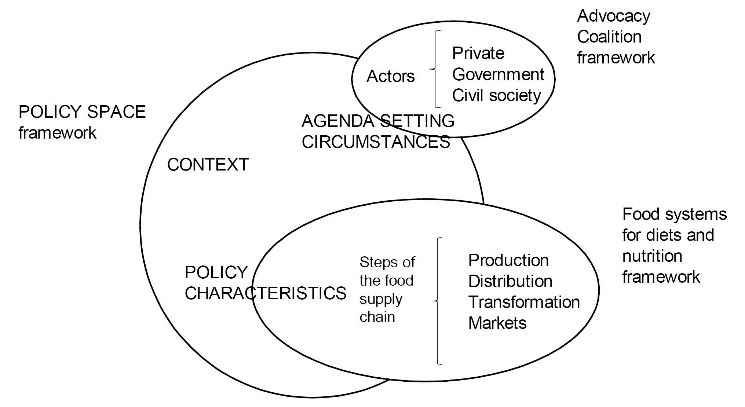
Frameworks Dimensions and Interrelations for Data Coding and Analysis.

As complementary analytical frameworks, the ACF^39^ and the food systems for diets and nutrition framework^14^ were embedded within the agenda setting and policy characteristics dimensions of the Policy Space framework (Figure 1). The ACF was chosen for its focus on actor dynamics relevant to policy change; it has also been previously used for nutrition policy analyses.^35^ The food systems framework identifies the different steps of the food supply chain: production, distribution (storage, transportation, food assistance programs), transformation (processing and packaging) and markets (commerce and trade) (Figure 1).^14^ The different steps of the food supply chain were used to guide the scope and definition of the food supply policies and also to guide the selection of appropriate sectors and policy documents. The identification of sectors was also based on the World Health Organization (WHO) recommendations for the action plan for the prevention of NCDs, which includes policy interventions that target the food supply.^41^

###  Data Sources and Study Informants

First, key policy documents with relevance to the steps of the food supply chain (production, distribution, transformation, and markets) were identified through searches of government websites of the agriculture, economic and health ministries. We selected policy documents that were published by government official channels such as the Official Gazette and government websites. Inclusion of policy documents was based on the programs with the most budget allocated within the 2019 and 2020 Sustainable Social Development Budgets^42,43^ and whose main program objective was related to the steps of the food supply chain. We triangulated the inclusion of policy documents with the programs mentioned by informants in the interviews to confirm that we had identified all relevant documents. We examined 19 selected policy documents. Information from the documentary data was extracted into a matrix in Excel^TM^, with columns based on the study frameworks, such as text related to the steps of the food supply chain, what drives policy and who coordinates the response. In addition, we extracted the stated policy objective and any mention of undernutrition, obesity or NCDs.

Second, we carried out 20 key informant interviews with stakeholders active in Mexico’s national food supply and nutrition policies (Table 1). The interviewees were drawn from across a range of actor types and three sectors: Agriculture, Economic and Health. Potential interviewees were identified based on government responsibilities (agriculture and health) and key nutrition institutions and alliances, followed by snowball sampling. Letters of invitation were sent to 27 heads of relevant government sectors and relevant nutrition institutions. Seven requests for interview were not responded to and 20 interviews were conducted. Interviews were semi-structured and investigated the respondent’s perspectives on nutrition problems, possible policy solutions in Mexico, drivers of policies in the food supply, and perceptions of the potential for an integrated food supply approach to malnutrition (including opportunities and constraints).

**Table 1:** Actor Type and Sector of Informants Interviewed

	**Agriculture (n)**	**Economic (n)**	**Health (n)**
Academia	3	2	3
Government	1	1	3
Private business, industry organizations		2	
Civil society, NGOs, international agencies	2	1	2
TOTAL (n = 20)	6	6	8

Abbreviation: NGOs, non-governmental organizations.

###  Data Analysis and Interpretation 

We drew on our theoretical frameworks to analyze both documentary and interview data and to identify constraints and opportunities in relation to increased consideration for nutrition in food supply policies. The analysis for process and interpretation of data was carried out in three phases. Interviews were transcribed and the data were first coded using NVIVO^TM^, based on predetermined codes and open coding (Table 2). The predetermined codes were informed by the study frameworks (Figure 1). The lead author coded the interviews and did the documentary review. Findings were reviewed iteratively by the research team throughout the analysis. Theoretical data saturation was determined when themes were repeated in interviews within sectors.

**Table 2:** Overview of Coding of the Study Frameworks

**Predetermined Codes**	**Open Coding**
Policy space: context (political, economic, nutritional), agenda setting (nutrition problem, possible solutions), policy characteristics (current economic policy priorities, current nutrition policy priorities). Food supply: production, distribution, transformation and markets (beliefs regarding the policies and situation of the steps of the supply chain).	Opportunities (specific ideas that informants note regarding how to improve the nutritional situation, with respect to food system policy). Actors interest (private sector, government, civil society interest with respect to food systems and nutrition).

 The second phase of the analysis looked for repetitive beliefs, ideas, concepts or/and narratives from most informants within the predetermined and open coded data in relation to potential constraints and opportunities, related to the research aim.

The third phase analyzed the open coded data of actors’ interests to identify key narratives and potential conflicts between actor groups (private, government, and civil society). This analysis deepened the understanding of the main findings underlying agenda setting circumstances for policy change by looking for differences in beliefs and ideas with respect to possible integration of nutrition priorities in food supply policy processes. The analysis for this open coding on actors interest was informed by the ACF,^39^ as the first approximation to political dynamics in terms of their beliefs and conceptualization of food policy related to nutrition.

 For interpretation of data regarding identification of main findings and opportunities, we looked for the most repetitive beliefs or ideas from informants for each of the codes, where repetition ranged from 6 to 17 informants. From the document data, we identified the dominant concepts and the current policy priorities relevant to food and nutrition. Analysis of interview and documentary data together was captured on a table organized by each dimension of the policy space framework for identifying the constraints and opportunities for policy change in light of the framework’s three key dimensions: the context, policy characteristics, and agenda setting. The results are presented below with reference to these key dimensions.

## Results

 We found that economic considerations were the primary concerns of the policy sectors that govern the food supply, and the focus was on food security related to food quantity rather than nutrition. Nutrition itself was not a policy consideration on its own: not in any of the agriculture or economy ministries we analyzed. However, we identified a number of opportunities to increase integration of nutrition on the multisectoral policy space in the key dimensions of the policy space framework: context, policy characteristics, and agenda setting. The following results are a synthesis of the policy space analysis in Mexico based on the policy document review and the informant interviews conducted in 2019 and 2020.

###  Context

 The context was characterized by a major shift in the politics in 2018 when a new president was elected from a left-of-center party. Food (agriculture and other food supply related industries) constitutes an important economic sector, and there was widespread recognition that nutrition is a growing public health and societal problem; however, food policies are currently not aligned with nutrition priorities.

 The main sectors governing the food supply in Mexico are the Ministry of Agriculture, Ministry of Economy, Ministry of Social Welfare, Ministry of Health, and the private sector. Each of these bodies contributes to the policy space differently. The Ministry of Agriculture is essentially dedicated to stimulating primary production. The Ministry of Economy regulates the trade and agro-industrial related policies of food products. Twelve international free trade agreements such as the United States-Mexico-Canada Agreement (USMCA renamed recently from NAFTA) have an external influence in Mexican agriculture, transformation, and trade policy. The Ministry of Social Welfare and Ministry of Health coordinate food distribution policies. A government agency linked to the Ministry of Health, the Federal Commission for Protection against Health Risks also regulates industry policy. The private sector participates in production, distribution, and transformation of food and markets. The main actors of the private sector influencing food policy are the agribusiness and the food processing industry. The food industry is organized in industrial chambers, which represents many companies of similar economy activity; for example, the corn dough and tortilla products industry.

Two main constraints were identified to integrate nutrition into the policy sectors that govern the food supply. The first is that the food supply policies respond mainly to economic priorities and are disconnected from nutrition priorities (Table 3). In the last 30 years, policies directed to the food supply have had the goal of improving productivity, exports, and economic growth. Nutrition policy priorities have focused on reducing the promotion and consumption of ultra-processed products high in sugar, refined carbohydrates, and fat. There seems limited effort to align food supply policies with nutrition policy. The second constraint identified was a historical focus on under- and overnutrition being addressed by separate policy sectors and documents. For example, the National Strategy for Obesity and Diabetes has been led by the Ministry of Health, while programs to address undernutrition have been led by the Ministry of Social Welfare. This historic fragmented view of nutrition is a constraint to building aligned policy efforts with nutrition at the core.

**Table 3:** Analysis of Policy Space to Integrate Nutrition as a Priority Into the Food Supply in Mexico

**Policy Space**	**Constraints**	**Opportunities**
*Context* (economic, political, nutritional)	A food supply which responds mainly to economic priorities (economic growth, exportation of food, productivity).	Goodwill by agriculture and economic informants in the discourse towards improving nutrition.
	Historical focus on under- and over- nutrition by separate policy sectors and documents (eg, National Strategy for Obesity and NCDs lead by Ministry of Health versus undernutrition lead by Ministry of Social Welfare).	Favorable political context to propose food supply policies with population nutrition at their core. The current government emphasizes more government intervention and downplays the role of the private sector in matters of societal concern.
		Dual burden of malnutrition (obesity and undernutrition) recognized as main problems of nutrition by informants. Informants recognized that nutritional concerns should be tackled from a food system perspective.
*Policy characteristics/ incentives on the steps of the food chain *(public and bureaucratic impact and potential conflict, resources and political support for implementation)	The nutrition assistance component of the conditional cash transfers program "PROSPERA" (program and budget) was eliminated in 2020, due to administrative inefficiencies.	Integration of nutrition criteria into existing policy programs, which are currently focused on staple foods (ie, support for production of corn, bean, rice, and wheat) but could be expanded to include other nutritious crops.
	Declining public budget in commercialization infrastructure and knowledge, contributing to limited connection between farmers and markets.	Open discussion for change between academia, government, and civil society around shortening food supply chains and developing local markets, to increase producer returns and create opportunities for small farmers.
	Vegetables and fruits are perishable foods. Improving storage and transport for V&F represents a significant investment for the private sector, NGOs, and government, and perceptions of benefit from this investment are limited.	NGOs and government initiatives (eg, school breakfasts) connecting fruit and vegetable small farmers to private and public procurements, main focus on training.
*Agenda setting circumstances* (nature of problem/advocacy, decision-making concerns)	Food security approach with main focus on food quantity and not diet quality advocated by the food supply coalition.	Recent political will for addressing food security and food self-sufficiency, with a new public body in 2019 "Segalmex" and an Agreement for Self-sufficiency.
	Perception by some informants that the government has focused on single issue aspects of the food supply with respect to nutrition (eg, front of pack labelling, SSB tax) instead of an integrated policy approach that brings together different sectors and experts.	In 2019 a multisectoral working group [GISAMAC] was established, with a newer narrative on food system transformation. Its vision is to create a healthy, sustainable, fair and competitive food system.

Abbreviations: NCDs, non-communicable diseases; V&F, vegetables and fruits; NGOs, non-governmental organizations; SSB, sugar sweetened beverages; GISAMAC, Intersectoral Food, Environment, and Competitiveness Group.

**Figure 2:**
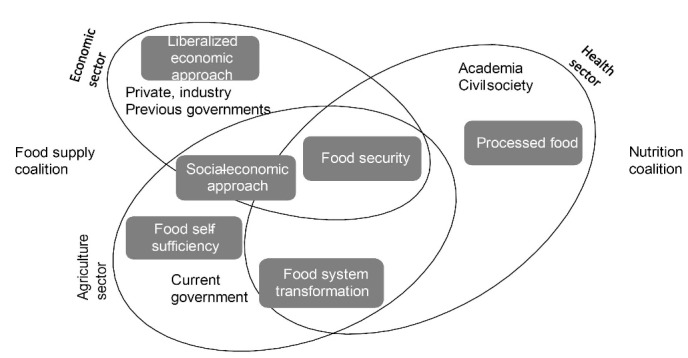
Visual Map of Different Concepts Propagated by Different Food Supply and Nutrition Actor Groups. The visual map illustrates a division among actor groups in how food security (at the center) is conceived: either oriented to food supply or to nutrition. The map can be read in two sides: the left one presents the food supply coalition as advocated by government economic and agriculture agencies and the private sector. The right side presents the nutrition coalition comprised mainly of civil society, government health sector and academia actors, who view food security as nutrition by reducing intake of processed food and increasing intake of nutrient-rich foods. On the left side, the economic sector with an existing liberalized economic approach wants no change to maintaining the status quo to food policy, while another group from the current government with a more social-economic approach brings a newer focus on food system transformation.

In this research, informants from government, civil society and academia identified in their narratives that the neoliberal economic approach of the previous governments as well as the private sector have generated opposition towards nutrition objectives (Figure 2). The private sector informants’ narratives continue to support the economic goals, which were described as: “the vested interests around the old institutional system mainly benefiting large transnational agri-food companies... but at this time the country has as a principle, having the interests of the people’s wellbeing ahead of it” [Government, Agriculture]. In other words, the current political ideology of the government is perceived as prioritizing those in society identified as disadvantaged compared with previous governments which prioritized private and corporate benefits. This narrative aligns with the recent change of policy documents that focuses on food policy attention on small and medium farmers to produce staple foods, replacing years of policy that supported large producers.

 The context presents opportunities, as we found a favorable political context to propose food supply policies with nutrition at their core with the current government which came to power in 2018. There was goodwill towards improving nutrition expressed by agriculture and economic informants in their discourse, which suggests an opportunity to position nutrition among the objectives in food supply policies. As an example, one informant stated, “We cannot have production policy incentives without considering consumption, and beyond consumption also comes health. I think that many people are not aware of this importance until it becomes clear the relation between food and health” [Academia, Economic]. This opportunity was also supported by recognition by 17 informants from the agriculture, economic and health sectors that malnutrition, obesity and undernutrition are the primary nutrition problems. Nine informants recognized that these nutritional concerns should be tackled from a food system perspective and not only by addressing determinants like education.

###  Policy Characteristics

 In the policy characteristics dimension, we found that increasing the productivity by small-scale farmers of “staple foods” such as corn, rice, wheat and beans was the main priority with most resources. However, there was little evidence that other nutrient-rich foods such as vegetables and fruits are given preference in food supply policies. We found that almost all public policies on food supply are aimed at directly influencing the step of primary production; however, much of the constraints and opportunities for policy change lie in commercialization: connecting small-farmers production to markets.

The key national policy documents influencing the food supply in Mexico can be divided into the different steps of the food supply chain depending on the main objective of the document: production, distribution, transformation, markets, and in addition, there are planning and finance documents (Table 4). While obesity and undernutrition are clearly identified as policy priorities in the planning policy documents, they were not as clearly identified in the implementation guidelines of the food supply chain policy documents. The planning documents “National Development Plan 2018-2024,”^44^ “National Agreement for Food Self-Sufficiency”^45^ and “National Agricultural Planning 2017-2030”^46^ do mention nutrition as a priority. They state that it is a national emergency to produce food in a sustainable and healthy way to combat malnutrition, banish undernutrition in children, contain and reverse the epidemic of obesity, and make effective the Constitutional Right to Food which is nutritious, sufficient and of quality. These planning policy documents are there to guide all efforts to produce food and address obesity, food insecurity and undernutrition. However, our review of the implementation guidelines of the food supply chain policy documents concerning production, distribution, transformation and markets revealed that undernutrition was only mentioned in the milk social supply program “LICONSA”^47^ and obesity in the “front of pack” labelling policy.^48^ Even though in planning documents obesity and undernutrition were identified as policy priorities, finance and food supply chain policy documents do not specifically mention malnutrition, and it is not evident that a wide range of nutrient-rich foods are given preference.

**Table 4:** Overview of National Policy Documents Affecting the Food Supply in Mexico

**Steps of the Food Supply Chain**	**Policy Document**	**Year Endorsed**	**Ministry Responsible**
Planning	National Development Plan 2019-2024	2019, April 30	Government/Chamber of Deputies
	Sectoral Program for Agriculture and Social Development 2019-2020	2019, August	Agriculture
	Decree establishing a Mexican Food Security Agency [SEGALMEX]	2019, January 18	Agriculture
	National Agreement for Food Self-sufficiency [Autosuficiencia alimentaria]	2019, February 8	Agriculture
	National Agricultural Planning 2017-2030 -- part 1	2017, September 14	Agriculture
Production	Wellbeing production program*	2019, January 23	Agriculture
	Rural Development program*	2019, February 28	Agriculture
	Guarantee prices program for staple food products in charge of Mexican Food Security*	2019, March 1	Agriculture
	Sowing life* [Sembrando Vida]	2020, March 30	Social Welfare
Distribution (storage, transportation, food assistance programs)	Rural Supply Program by DICONSA S.A. from C.V*	2019, March 1	Agriculture
	Milk Social Supply Program by LICONSA S.A. de C.V.*	2019, March 1	Agriculture
	PROSPERA social inclusion program*	2019, February 28	Social Welfare
	Community Health and Wellness Program* [School breakfasts]	2019, December 28	DIF
Transformation (processing and packaging)	Tax on sugar-sweetened beverages and "non-staple foods with high caloric density"	2013, December 11	Government/Congress of the Union
	Front of pack labelling	Initiated in 2014, modified March 2020	Health, Economy
Markets (commercialization and trade)	Social and sustainable agromarkets program*	2019, March 21	Agriculture
	USMCA	Free trade initiated 1990, modified to USMCA in 2018	Economy
Finance	Budget of the PEC for Sustainable Rural Development 2019	2018, December	Chamber of Deputies/CEDRSSA
	Budget approved for 2020 of the Special Concurrent Program [PEC] for Sustainable Rural Development	2019, December	Chamber of Deputies/CEDRSSA

Abbreviations: CEDRSSA, Center for Studies for Sustainable Rural Development and Food Sovereignty; USMCA, United States-Mexico-Canada Agreement; DIF, Health/National System for Integral Family Development; PEC, Special Concurrent Program. *Implementation guidelines of public programs published in Official Gazette, with budget attached to them.

The document review and informant narratives revealed constraints to integrate nutrition as a priority. This was evident in the policy priorities and budget focus on productivity, the declining budget supporting commercialization infrastructure and knowledge to connect the production of small-farmers to markets, and the elimination of the food assistance program “PROSPERA” (Table 3). Production documents^49-51^ priorities were mainly to increase production of small farmers focused on “staple foods” such as corn, rice, wheat, and beans. But support to promote production of a wide range of nutrient-rich foods as nutritious crops, such as vegetables, was not evident. The budget focus was mainly on production programs, and just one program in 2019, the “Agromarkets”^52^ program, was designated for commercialization to connect farmers to markets, which included support for storage infrastructure and training for agricultural competitiveness. Unfortunately, in 2020, the “Agromarkets” program was eliminated. Reducing focus on commercialization makes it difficult for people to have access to a range of nutrient-rich foods. The food assistance component of the “PROSPERA”^53^ program that addresses undernutrition was eliminated in 2020. Some interviewees mentioned this budget elimination of “PROSPERA” was not a good decision. As one informant mentioned “the conditional transfers to the nutrition outcome was not bad, and we haven’t finished the undernutrition problem. It can grow back” [Academia, Health].

 One of the opportunities identified for strengthening nutrition within the current policy actions is to integrate nutritional criteria into the agricultural production and distribution policies in the selection of crops and food to support. Interviewees mentioned that the main objective of production policies had been in increasing production of large-scale farmers, and, in 2019, the newly elected government embarked on series of programs to support small-scale farmers. In terms of choosing crops to support, since 2019, production programs have been oriented to “staple foods,” and six informants reported that in these policies fruits and vegetables are absent. Production programs such as “Wellbeing production” and “Guarantee prices” could be tailored to better support nutrition by integrating nutrition criteria in the selection of crops to support and to integrate vegetables and fruits among crops to be supported.

The distribution policies most mentioned by interviewees were “LICONSA,”^47^ “DICONSA”^54^ and “school breakfasts,”^55^ which are food assistance programs (Table 4). The opportunity mentioned by policy-makers concerning distribution policies is to include local and fresh foods in their offerings. One informant stated, “The challenge is to include fresh and regional foods and not only provide a collection of industrialized products, many of them with low nutrient value and high in calories” [Government, Health]. Some ideas mentioned were to review the way in which public procurements are implemented for public purchases of food (eg, for “school breakfasts”) because the procedures are designed for large companies and not necessarily for purchasing from small local farmers. In the public procurement system for food purchases, there is the opportunity to include vegetables and fruits from local farmers with the objective to improve the nutritional value of food assistance programs.

 Another opportunity mentioned by informants was to increase financial and technical support for commercialization of crops from small-scale farmers, in which nutrient-rich foods such as vegetables and fruits could be relevant from a nutritional point of view. Financial support could be allocated to post-harvest handling, storage infrastructure and transport. Mainly two strategies were proposed: shortening food supply chains and developing local markets. Five informants proposed shortening food supply chains to bring the small farmer closer to the consumer by decreasing distance between production and consumption and the reduction of intermediaries. The other strategy mentioned was developing local markets in order to connect small farmers to consumers. According to one interviewee, both these objectives could be achieved, proposing that the current policy of supporting the income of small farmers through the “Wellbeing production” program could be combined with a public policy for the development of local markets. As he stated, “By having a shorter supply chain, the producer would increase their return, which would stimulate production” [Academia, Economic].

 Informants mentioned that the development of local markets implies having a policy of public goods and infrastructure that allows greater capacity to link small farmers with consumers in nearby markets. This possibility of developing local markets cannot take place as a business decision by small farmers alone. The decision will have to be made with the three levels of government: federal, state and municipal in collaboration with non-governmental organizations (NGOs) and international agencies. This open discussion for change around shortening supply chains and developing local markets is an opportunity for increasing consideration of nutrition objectives in food supply policy-making.

 Aligned with this opportunity to support commercialization, we found that training in business management for small farmers was important to enhance production of a wide range of nutrient-rich foods and connect them to procurements and markets. Three informants mentioned existing interventions connecting fruit and vegetable small farmers with private and public procurement. For example, an NGO is supporting small fruit and vegetables farmers to link their crop production to government procurement or private purchases by large companies and supermarkets. The support is mainly technical with a focus on training farmers to develop business capacities, in which profitability and understanding procurement of public distribution programs and purchase requisitions of large companies are important. However, informants mentioned as a constraint that vegetables and fruits are perishable foods. Improving storage and transport would represent a significant investment for the private sector, NGOs and government, and perceptions of benefit from this investment are limited.

Potential conflicts between intermediaries that currently connect production with markets, and the government policy instruments that mainly focus on production, were mentioned by academia and civil society informants. These intermediaries include people that connect production with retailers. The motivation for bypassing the intermediaries and directly connecting the small farmers to the market is to improve the profit margins of the farmers, but implementation would potentially conflict with the interest of the intermediaries. An alternative means identified in the policy instruments of the current government that provides cash transfers to help the small farmers^49,50,56^ is training. Civil society and academia informants reported that part of the solution should be on training and in building capacities in reaching the “big buyers.” “The big buyers… they have these very difficult purchasing policies and the small ones do not even know what they are asking for, and therefore they have to deliver their production to the intermediary…” [Civil society, Agriculture]. However, the training and building capacities seemed to have less political traction because it does not offer political support in the way agricultural cash transfers do.

###  Agenda Setting Circumstances

Our analysis found that there were diverse perspectives among informants regarding the potential role of food supply policies in addressing malnutrition. The document reviews and interviews suggest that previous food security narratives of quantity that are already being addressed by the agriculture sector are encountering a newer policy narrative of food system transformation introduced by academia and civil society international actors. This newer narrative on food system transformation, already on the public agenda through a multisectoral working group, represents an opportunity to shift the existing food security narrative away from a main focus on food quantity and food self-sufficiency and towards a concurrent focus on the diet quality needed for population nutrition improvement (Figure 2).

Food security was the dominant concept found in the informant interviews and documents narratives that related nutrition to food policy. The main objective related to nutrition of food policy was to contribute to food security, according to nine informants from the agriculture, economic, and health sectors. Food security was represented differently by two main coalitions of policy actors; one with its main emphasis on food supply, and one on nutrition (Figure 2). There was however, some overlap in these coalitions, (for example, the current government agriculture actors are envisioning both ways). The policy documents and informants’ narratives indicate that the actors on the food supply coalition broadly are maintaining a focus on food security related to the provision of enough quantity of food, of calories, and producing enough staple foods; but little consideration is given to diet quality. In contrast, those in the nutrition coalition are bringing a focus on diet quality, including reduction of processed food and a transformation of the food system.

A dichotomy of framing of food security was evident when choosing policy actions. Informants from the food supply coalition linked food security policy to the concept of food self-sufficiency but with two different approaches (Figure 2). Six informants mainly from the agricultural sector mentioned increasing internal production and decreasing importation of food of foreign origin, at least for the “staple foods” like corn, beans, wheat, rice, milk and meat products. This self-sufficiency narrative is consistent with the objectives of the planning policy documents and is more aligned with the socio-economic approach of the current government.^44,45,57,58^ In contrast, two informants involved with the private sector criticized this self-sufficiency concept and have a more liberal economic approach to food security. Their discourse included denigrating ‘self-sufficiency’ as a policy objective as being inconsistent with a competitive world and promoting trade as achieving a more efficient food supply. They prioritized designing a policy in which regions are dedicated to better production and depending on profitability and market potential. This more liberal economic take was also found in policy documents such as the trade agreement between USMCA.^59^

Five informants across all coalitions identified that a key constraint to integrating nutrition into food policy was that the government strategy to address malnutrition has focussed on specific interventions, without an integrated policy (Table 3). The most common intervention discussed was the implementation of food labelling,^48^ mentioned by 12 informants, all from the health sector and one third of the agricultural and economic sector informants. For example, one stated, “Food labelling is an example of a public policy instrument, without having a public policy to work on” [NGO, Health]. This lack of integration may have been a result of a wider disconnect evident within the nutrition coalition, in which actors were found to be split between an internationally supported actors group believing in policy action to reduce processed foods for obesity prevention, and a group of national actors focused on food security, hunger and undernutrition.

 Some recent evolution was evident within the food supply coalition. The current government has a more socio-economic approach to food policy, in contrast to years of a market-based approach of previous governments. The current government has recognised the need for food policy to strike some balance between promoting economic objectives against nutritional and environmental ones. There needs to be a balance between a market-based approach pursued by the economic sector and previous governments and the current government’s approach that uses policy and regulation to promote integrated policy and organizational transformation.

 The most mentioned opportunity for agenda setting by informants was the actual political will to change through a multisectoral group that brings a newer narrative of food system transformation through a vision to build a healthy, sustainable, fair, and competitive food system. There is a window of opportunity with the inter-institutional group called Intersectoral Food, Environment, and Competitiveness Group (GISAMAC, for its initials in Spanish) which is working on building a policy proposal, and was mentioned by eight informants. The group includes different institutions of the federal government, academia, and civil society organizations.

 In the newer narrative on food system transformation with the vision to build a healthy, sustainable, fair, and competitive agro food system, the informants discussed the relationship between terms of the vision. It is interesting to note how actors from different sectors conceptualized the relationship between the terms “sustainable” and “healthy” differently. The agriculture informants mentioned how modern technologies, specifically the use of insecticides, are affecting the environment and deteriorating the nutritional content of food. In contrast, the health informants related sustainability with a lower carbon footprint in the environment of local production and improvement of nutritional quality due to less food processing. In terms of what constitutes healthy, informants in the agriculture and health sector saw fruits, vegetables and legumes as foods that present health benefits, and sugar was perceived as a food of health concern. Increased consumption of meat was seen as healthy, especially for population at risk of undernutrition, and nuts were not mentioned. The term “fair” was also explained by the informants. Fair meant an equitable distribution of benefits, related to fair agriculture in which the small farmer also wins. An example mentioned by an informant was the “fair price” that SEGALMEX pays to small farmers in the public procurement for food. This “fair price” payment is also mentioned in the “Guarantee prices” policy document for the procurement of milk. The challenges mentioned for the GISAMAC is to create an integrated long-term vision and food policies with a comprehensive implementation plan, and not merely collect together the different actions that each institution is already doing.

## Discussion

This policy analysis study aimed to identify opportunities to integrate nutrition as a priority into the food supply policy space in Mexico. We used Policy Space Analysis^38^ and embedded the ACF^39^, and the steps of the food chain of the food systems framework^14^ to contribute a primary analysis of the contextual, political and policy factors that creates constraints and opportunities for the food supply chain policy process to integrate nutrition priorities. We applied these frameworks to Mexico as a case study using document analysis and key informant interviews. We make it possible to understand in a LMICs context what it would look like to think differently about food systems, in line with international recommendations on food system transformation to deliver healthy diets.^8,14,19^ As in other LMICs, we found that economic considerations such as productivity and exportation were primary concerns in the food supply, despite a competing view from other sectors (health) that food security ought to consider diet quality over food quantity.

When analyzing the current policy space that influences the Mexican food supply, we found a variety of opportunities for nutrition integration. First, the political context is favorable to propose food supply policies with nutrition at their core. Second, in the policy characteristics, there is an opportunity to develop strategies for agricultural production and food distribution to improve their impact on nutrition. For example, nutritional criteria could be integrated into the selection of “staple” crops and foods that receive support by government. Third, there is the opportunity to increase financial and technical policy support for the domestic commercialization of vegetables and fruits. Nutrition advocacy could highlight the existing initiatives of small farmers to connect fruit and vegetable production with procurements and markets. Fourth, there is the opportunity to advocate in the public agenda for a long term food policy within the multisectoral group called GISAMAC that could provide wider recognition of nutrition and sustainability as key aspects of food security.^60^

Our findings are consistent with previous research that suggests that food policy is focused on economic priorities; the agriculture and economic sectors that are responsible of the food supply governance are dissociated from nutrition. In Latin America and in Africa in the 1990s, liberalization has shifted discourse and policies over time towards more economic priorities in food policy.^61,62^ In India, nutrition’s role is limited in part due to economic paradigms that have shaped discourse on food policy.^3^ Such policies have supported a focus on export-led agriculture, similar with what we found in Mexico, particularly since the NAFTA was initiated in 1994. However, the current political context in Mexico suggests opportunities to consider nutrition in food policy. The current political party in power places more focus on state interventions and decreasing private sector activities. We suggest that this new context presents the opportunity to rebalance economic sector objectives with health objectives that includes nutrition priorities.^63,64^

It was evident from the policy documentation and interviews that nutrition-relevant aspects of food policy in Mexico were often narrowly conceived of as food security, and this is characterized by food quantity, providing sufficient calories, and limiting the focus on dietary quality. This narrowing of the food policy to food security also has been seen in Zambia with actor coalitions focused on food security and on nutrition,^35^ and in South Africa, with the addition of a coalition focused primarily on economic objectives.^65^ Our findings are also consistent with the historical paradigm that was found in Ghana in relation to policy mandates, with a separation of responsibilities for food (agriculture) and nutrition (health).^62^ In Mexico as in Ghana, the agriculture sector is responsible for ensuring national food security, with a production paradigm focused on food sufficiency, while the health sector mandates nutrition priorities, with little attention to the food supply. In considering the opportunities for change to food policy in Mexico, it is also important to recognize that the current dominant approach of increasing productivity or food assistance programs has been shown to be ineffective to increase food security.^66,67^

In this study, we see a timely opportunity to reduce this policy dichotomy (food supply vs. nutrition) in the case of Mexico with the current working group on food policy. Our analysis indicates this is a moment in the policy space to take advantage of the current vision on the public agenda to have a food system transformation which integrates agricultural and nutrition strategies to improve food systems with the objective of better population nutrition. Developing such strategies would support the food system change agendas and is consistent with the literature that examines concerns of dietary quality and not simply a narrow economic approach for food security.^14,15,19,20,60^ In Mexico, the multisectoral working group GISAMAC represents an existing vision on the public agenda that presents an opportunity to integrate nutrition in the food system transformation.

Resistance to change from existing beneficiaries of food policy and fragmentation across government agencies also creates barriers to the integration of nutrition considerations in food policy. The private sector, as large-scale agricultural producers and as the intermediaries in Mexico, mirrors the dynamic seen in Latin America more broadly, where the (large) food industries are the existing beneficiaries and have been resistant to change because of their interest in maintaining a status quo.^68^ This resistance has mainly been enacted through lobbing government during the development of national health policies.^69,70^ Compounding this fragmentation are diverse priorities amongst the nutrition community, both in Mexico and elsewhere, with separate groups concerned about obesity, diet-related NCDs, undernutrition, hunger, and food security.^35,71-73^

Malnutrition – obesity and undernutrition - remains an issue in Mexico as in other LMICs. The risk factor of diet quality^5,6^ cannot be overcome through the food security agriculture and economic sectors. While the different sectors – agriculture, economy and health - have largely worked separately on their own issues in Mexico,^68^ informants in this research professed goodwill towards improving nutrition as a means to lessen malnutrition. Our informants recognized that nutritional concerns should be tackled from a food system perspective, which can be an entry point for change.

###  Study Strengths and Limitations

 The findings presented in this study are from a policy space analysis which provides context-specific information for pursuing an integrated food supply policy approach to nutrition in Mexico. A key contribution of this study is providing insights into both content and strategy opportunities for improving a multisectoral approach to nutrition. We note that the study has some limitations. It is limited by the number of interviews we conducted in each sector, particularly considering the complexity of the food supply chains and malnutrition problem, in which the food chains are dynamic subsystems that interact with other sectors and systems. However, we were able to reach high-level decision makers with relevant expertise in most cases. The conceptual frames used in this study guided us to make binomials and categorizations such as supply and demand, food supply chain policy steps, and under- and over-nutrition. These binomials and categorizations were made in order to understand the phenomenon, but a challenge remains to integrate them with a food system perspective. In addition, the first author’s positionality as a nutritionist could potentially be influencing the perspective of the results. On the other hand, she is an outsider of the food supply policy space with the advantage of the unfamiliar with food supply beliefs and priorities. The study is also limited because it was done in a point in time of a political party in charge and policy documents which were changing. However, the current timing of this study becomes even more relevant with the outbreak of COVID-19 which is demonstrating the fragility of the food systems for food security and nutrition.

## Conclusion

 Solving malnutrition – both over and undernutrition – is a public policy goal for many LMICs, and Mexico is no exception. Yet, integrating nutrition into national food policy is influenced by opportunities and constraints that must be understood to help inform food and health policy-makers regarding which policy actions can be undertaken for policy change. Our policy space analysis revealed the tensions between different national sectors (economy, agriculture and health) through this exploration of different actors’ beliefs regarding food policy and nutrition. Our research also identifies opportunities for specific points of change in the existing food policies in Mexico, focusing on food systems supply chains. Taking reparative action potentially supersedes domains of actors’ influence and could provide a meeting point with a new food systems transformation narrative.

## Acknowledgements

 This manuscript is drawn from a doctoral dissertation being prepared for the Master´s and Doctorate Program in Medical and Health Sciences of the National Autonomous University of Mexico. In addition, Dr. Karen Englander an English-speaking science editor provided assistance with English language editing.

## Ethical issues

 The research was previously approved by the Ethics Committee of the National Autonomous University of Mexico [PMSCMOS/CEI/007/2019].

## Competing interests

 GC Cervantes reports other from National Council for Science and Technology in Mexico (CONACYT), during the conduct of the study.

## Authors’ contributions

 All of the authors listed made substantial contributions to the manuscript and qualify for authorship, and no authors have been omitted. LDA directed the research, and reviewed the manuscript critically. GC conducted research, compiled data, drafted the manuscript, and coordinated revisions. AMT provided insight into scope, contributed to data analysis and interpretation and reviewed the manuscript critically. LGO and CPF reviewed the research proposal and manuscript critically.

## Funding

 This work was supported by the National Council for Science and Technology (CONACYT) in Mexico [grant number CVU 806118].
